# Spontaneous cholecystocolic fistula: an uncommon complication of chronic cholecystitis

**DOI:** 10.1002/ccr3.1215

**Published:** 2017-09-29

**Authors:** Vishakha Agrawal, Utsav Joshi, Sujan Manandhar

**Affiliations:** ^1^ Maharajgunj Medical Campus Institute of Medicine Tribhuvan University Kathmandu Nepal; ^2^ Department of Surgery Institute of Medicine Tribhuvan University Kathmandu Nepal

**Keywords:** Cholecystectomy, cholecystocolic fistula, cholelithiasis, chronic cholecystitis

## Abstract

Cholecystocolic fistula, a rare complication of long‐standing gallstone disease, is a diagnostic challenge owing to nonspecific clinical presentation and lack of accurate preprocedural diagnostic modalities. In case of incidental discovery of the fistula during the surgical procedure, excision of the fistula with repair of the colonic defect is imperative.

## Introduction

Biliary enteric fistulas are the most common kind of internal biliary fistulas, of which cholecystocolic fistulas are the second most common type (6.3–26.5%) 1, 2, 3, 4. Gallstones are the predominant pathology responsible for causing cholecystocolic fistulas. Cholecystocolic fistula is a diagnostic challenge owing to the absence of hallmark signs clinically and on preprocedural imaging. Even though more surgeons are opting for minimally invasive surgery, traditional method of open cholecystectomy with fistula excision remains the mainstay of treatment in challenging cases and in cases with difficult access.

We present a case of a middle‐aged female, who was found to have cholecystocolic fistula intraoperatively while being operated laparoscopically for cholelithiasis.

## Case Presentation

A 52‐year‐old hypertensive female patient presented to the outpatient department of a university hospital with a history of intermittent right upper quadrant abdominal pain for 4 years which had been increasing in severity for the past 1 month. This was not associated with fever, vomiting, or jaundice. She did not give any history of weight loss, and her bowel habit was also normal. She had undergone emergency appendectomy 1 year back for acute appendicitis. On examination, there was no icterus and she was afebrile. Examination of the abdomen did not reveal tenderness, and there was no guarding, rigidity, or rebound tenderness.

The hemogram revealed a hemoglobin level of 10.8 g% with packed cell volume of 33.8%; a white cell count of 5750/mm^3^ with 75% neutrophils, 21% lymphocytes, and 3% eosinophils; and a platelet count of 109,000/mm^3^. Liver function test was also normal (total bilirubin level: 17 *μ*mol/L, direct bilirubin: 5 *μ*mol/L, aspartate transaminase: 17.0 U/L, alanine transaminase 13.0 U/L, alkaline phosphatase: 214.0 U/L, amylase: 63.0 U/L, gamma‐glutamyl transpeptidase: 54.0 U/L and lactate dehydrogenase: 301.0 U/L). Plain abdominal radiograph revealed normal findings. Abdominal ultrasonography revealed a calculus measuring approximately 17.2 mm within the lumen of the gallbladder. A provisional diagnosis of symptomatic cholelithiasis was made.

**Figure 1:**
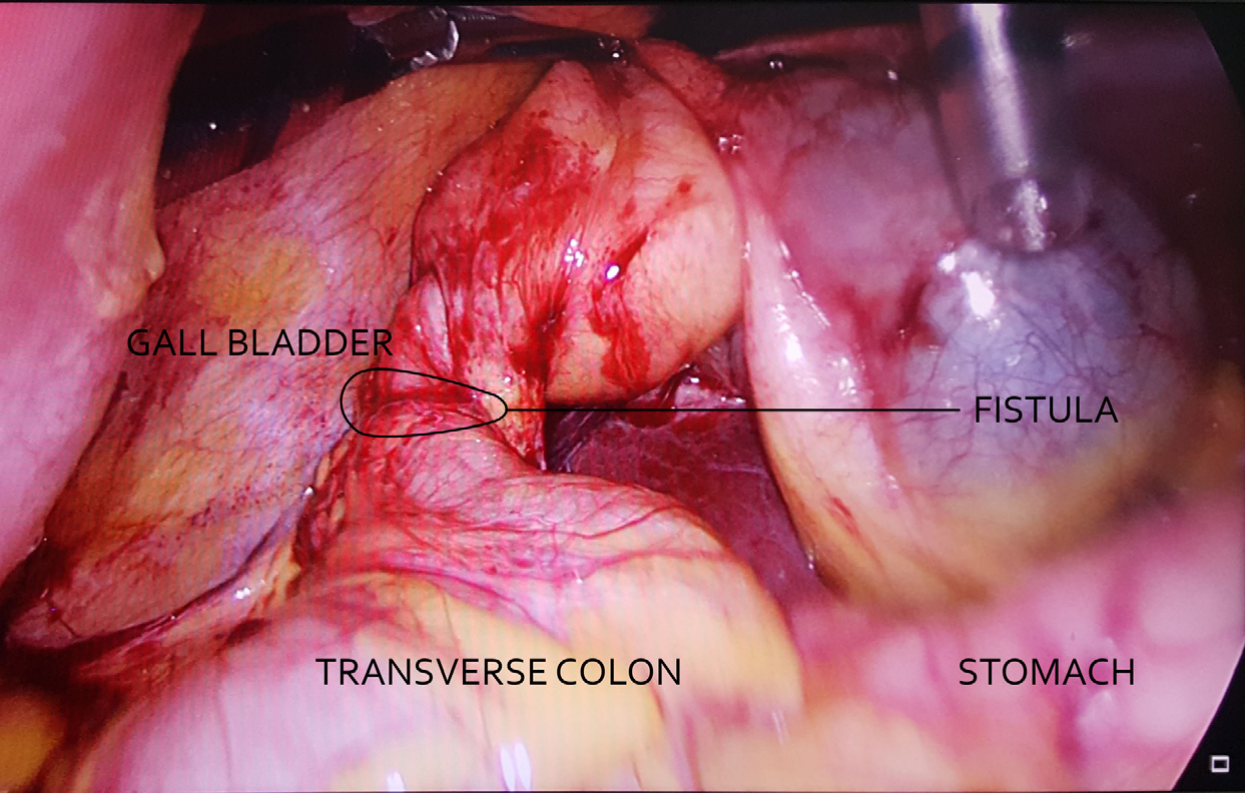
A cholecystocolic fistula is evident between the fundus of the gallbladder and the transverse colon.

**Figure 2:**
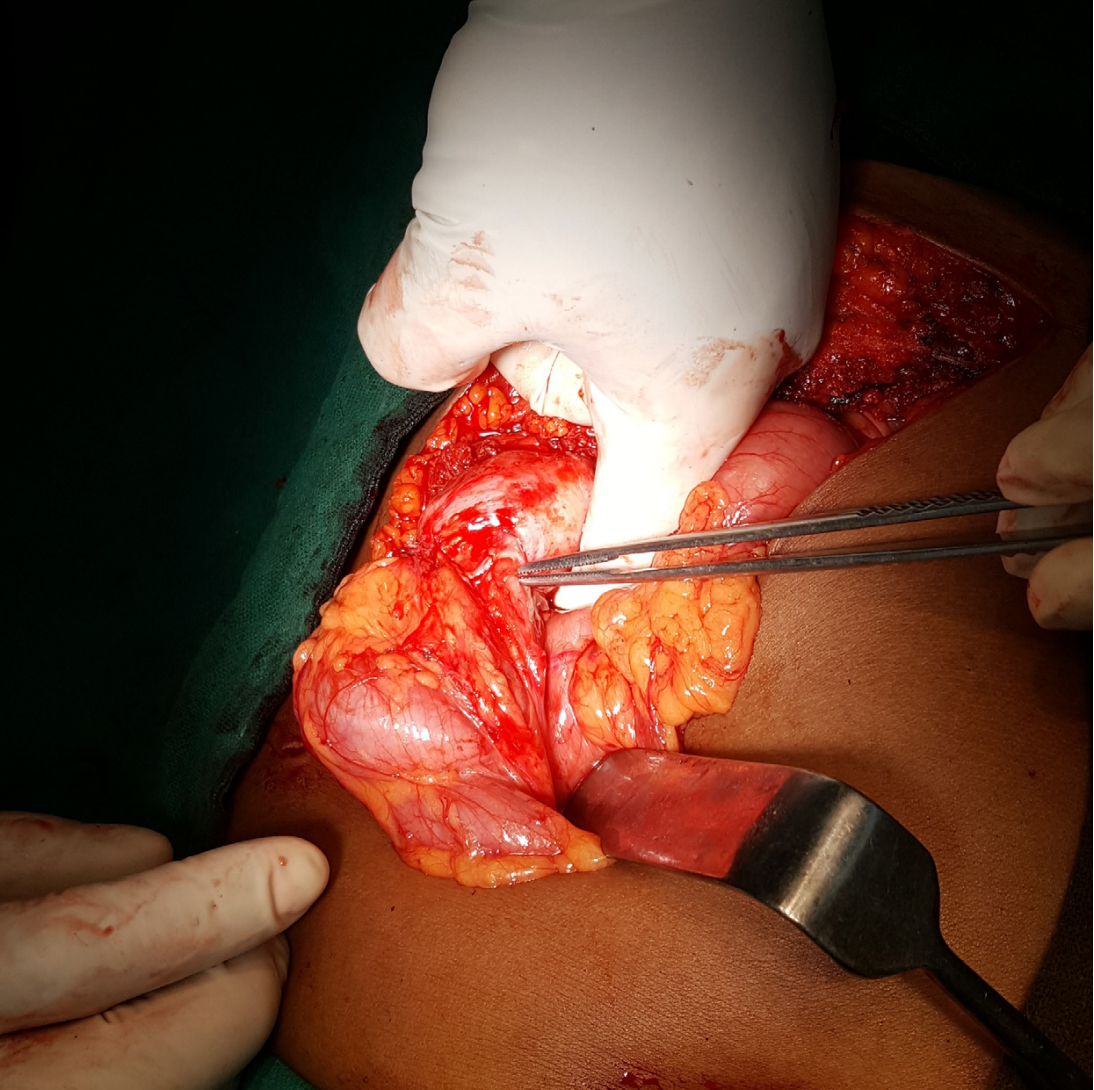
A cholecystocolic fistula. The location is denoted by the position of the forceps and the gloved finger.

**Figure 3:**
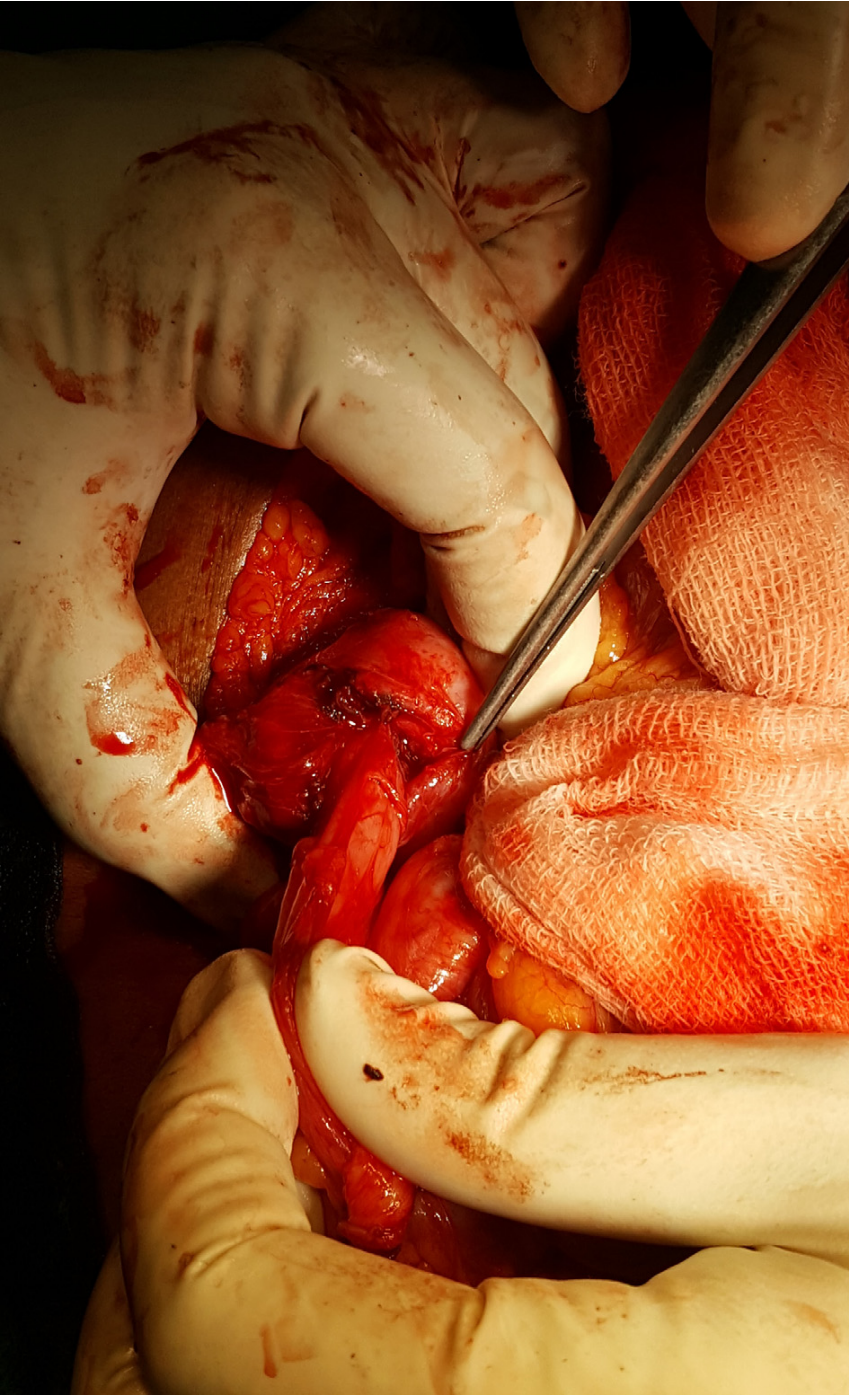
Excision and closure of the cholecystocolic fistula are shown.

The patient was planned for laparoscopic cholecystectomy. During laparoscopy, a fistulous tract was evident between the fundus of the gallbladder and the transverse colon. Hence, the initial laparoscopic approach for cholecystectomy was converted to open via a right subcostal incision. The cholecystocolic fistula was excised along with primary repair of colonic defect. The Calot's triangle was dissected, and the gallbladder was separated off the gallbladder fossa. The cystic artery and the cystic duct were isolated, ligated, and cut. A 2 cm × 2 cm large calculus was noted in the fundus along with two smaller calculi within the gallbladder lumen (Figs 1, 2, 3).

The patient's hospital stay was uneventful, and she was discharged without any complications on the third postoperative day. Histopathological examination of the specimen revealed lympho‐plasmacytic infiltrates in the lamina propria and fibrosis in the subserosa, features suggestive of chronic cholecystitis.

## Discussion

Between July 2015 to February 2017, 686 patients underwent cholecystectomy in our department, with only one patient being diagnosed with a cholecystocolic fistula. Here, we describe this case of cholecystocolic fistula as a rare, yet, important complication of gallstone disease. The vague and variable clinical presentations and the lack of highly sensitive preoperative diagnostic tools make it difficult to clinically suspect the presence of cholecystocolic fistula and hence pose a significant diagnostic dilemma.

Biliary enteric fistulas have an incidence of 0.9% of biliary tract surgeries for nonmalignant causes 4. In majority of the cases, long‐standing cholelithiasis and chronic cholecystitis predispose to the development of the fistula that most commonly develop between the gallbladder and duodenum (55.9–77%) followed by the transverse colon (6.3–26.5%) and the stomach, which account for the remainder 1, 2, 3, 4. However, Crohn's disease, abdominal trauma, and malignancy of the biliary tract, bowel, and the head of pancreas have also been reported to be associated with the development of the fistula 3. Cholecystocolic fistulas have a female preponderance and are commonly found in the elderly, with a mean age of 68.9 years 5.

Savvidou in 2009 proposed a triad of pneumobilia, chronic diarrhea, and vitamin K malabsorption to be pathognomonic for cholecystocolic fistula 6. However, due to the lack of studies pertaining to the sensitivity and specificity of this triad, only diarrhea should be considered as the key symptom of cholecystocolic fistula. While chronic diarrhea is the most common symptom in nonemergency presentation (71%), it was absent in our patient 7. Nevertheless, patients may also present with abdominal pain, jaundice, fever, nausea, vomiting, steatorrhoea, and weight loss. In acute conditions, cholecystocolic fistulas are responsible for 4.8% of gallstone ileus, but the patients may also present with massive bleeding and liver abscess 5, 8. Be that as it may, a substantial proportion of the cases remain asymptomatic and are only discovered incidentally intraoperatively, as in our case.

Owing to a wide array of nonspecific symptoms and inefficient diagnostic modalities, cholecystocolic fistula has a low preoperative diagnosis rate (<10%) 1. According to studies performed in large series of more than 10,000 patients undergoing cholecystectomy, the incidence of the inadvertent discovery of cholecystocolic fistula during the procedure is reported to be 0.03–0.13% 1, 2, 4. Even with the advent of newer imaging techniques, not one tool has shown promising results in diagnosing cholecystocolic fistula. Barium enema has led to a diagnosis in majority of the cases preoperatively, but is shown to have low sensitivity 5. Pneumobilia is usually absent in plain abdominal radiograph, but can be visualized in computed tomographic (CT) scan 9. Endoscopic retrograde cholangiopancreatography (ERCP) is one tool that can aid in both diagnosis and successful management of the fistula, but has shown variable results 5, 9. Investigations like ERCP, CT scan, and colonoscopy which are not only invasive or expensive, but also lack sufficient studies pertaining to their sensitivity and specificity are not performed routinely in patients presenting with diarrhea and abdominal pain, unless complications are suspected. In such cases, abdominal ultrasonography, which is inexpensive, noninvasive, is considered as the first line of radiological investigation.

Cholecystectomy with closure of fistula has been considered as the standard treatment for the management of cholecystocolic fistula and can be reproduced laparoscopically in uncomplicated cases. Although laparoscopic approach may decrease the length of hospital stay, but its association with higher conversion rates (55%) and longer operating time may be a cause of concern in elderly patients with coexisting comorbidities which are present in 45% of the patients 2, 3. In complicated cases, the surgeons may have to switch from laparoscopic cholecystectomy to an open, custom‐made procedure that may additionally involve colonic resection.

In the presented case, the patient was initially diagnosed to have symptomatic cholelithiasis in isolation. The clinical presentation and preoperative investigations did not suggest the presence of cholecystocolic fistula. However, during laparoscopic cholecystectomy, the operating surgeons visualized a fistula connecting the fundus of the gallbladder and the transverse colon. Failure to detect such fistulas during operation can be devastating due to risk of division of fistula and colonic perforation. In situations like these, when there is an incidental evidence of fistula during surgical procedure, the fistulous tract should be obliterated. The obliteration of the fistula removes the theoretical risk of cholangitis that can occur because of colonic bacterial translocation through the tract into the biliary system. Moreover, laparoscopic procedure should be converted to open if there is difficulty in accessing the fistula site, as in our case.

## Conclusion

Cholecystocolic fistula is an uncommon complication of cholelithiasis. Even though the clinical symptoms and signs are nonspecific and there are no diagnostic modalities specific enough to make a diagnosis preoperatively, clinicians should be aware of its possibility in patients with long‐standing cholelithiasis and chronic cholecystitis. Once the fistula is detected intraoperatively, it is imperative to excise the fistula, either by laparoscopic or by open approach, whichever feasible.

## Conflict of Interest

The authors declare that they have no competing interests.

## Authorship

VA: involved in conception and design, acquisition of data, and analysis and interpretation of data; drafting the article; and final approval of the version to be published. UJ: involved in conception and design, acquisition of data, and analysis and interpretation of data; drafting the article; and final approval of the version to be published. SM: involved in conception and design; critical revision of the article; and final approval of the version to be published.
